# Development of a New Monomer for the Synthesis of Intrinsic Antimicrobial Polymers with Enhanced Material Properties

**DOI:** 10.3390/ijms160820050

**Published:** 2015-08-24

**Authors:** Florian Brodkorb, Björn Fischer, Katrin Kalbfleisch, Oliver Robers, Carina Braun, Sophia Dohlen, Judith Kreyenschmidt, Reinhard Lorenz, Martin Kreyenschmidt

**Affiliations:** 1 Institute of Construction and Functional Materials, University of Applied Sciences Münster, Stegerwaldstraße 39, 48565 Steinfurt, Germany; E-Mails: bjoern.fischer@fh-muenster.de (B.F.); katrin.kalbfleisch@fh-muenster.de (K.K.); o.robers@fh-muenster.de (O.R.); rlorenz@fh-muenster.de (R.L.); martin.kreyenschmidt@fh-muenster.de (M.K.); 2 Institute of Animal Science, University Bonn, Katzenburgweg 7-9, 53113 Bonn, Germany; E-Mails: cbraun@uni-bonn.de (C.B.); sdohlen@uni-bonn.de (S.D.); j.kreyenschmidt@uni-bonn.de (J.K.)

**Keywords:** monomer, antimicrobial polymer, biocide, intrinsic antimicrobial

## Abstract

The use of biocidal compounds in polymers is steadily increasing because it is one solution to the need for safety and hygiene. It is possible to incorporate an antimicrobial moiety to a polymer. These polymers are referred to as intrinsic antimicrobial. The biocidal action results from contact of the polymer to the microorganisms, with no release of active molecules. This is particularly important in critical fields like food technology, medicine and ventilation technology, where migration or leaching is crucial and undesirable. The isomers *N*-(1,1-dimethylethyl)-4-ethenyl-benzenamine and *N*-(1,1-dimethyl-ethyl)-3-ethenyl-benzenamine (TBAMS) are novel (Co-)Monomers for intrinsic anti-microbial polymers. The secondary amines were prepared and polymerized to the corresponding water insoluble polymer. The antimicrobial activity was analyzed by the test method JIS Z 2801:2000. Investigations revealed a high antimicrobial activity against *Staphylococcus aureus* and *Escherichia coli* with a reduction level of >4.5 log_10_ units. Furthermore, scanning electron microscopy (SEM) of *E. coli.* in contact with the polymer indicates a bactericidal action which is caused by disruption of the bacteria cell membranes, leading to lysis of the cells.

## 1. Introduction

The use of biocidal compounds in polymers is steadily increasing because it is one solution to the need for safety and hygiene. A compound that kills or inhibits the growth of microorganisms is referred to as a biocide. Potential fields of application include for example hospital surfaces/furniture, surgery equipment, health care products or food packaging. Two different categories of antimicrobial materials are differentiated. The biocide can be either temporarily trapped in the polymeric matrix or permanently attached to the backbone of the polymer. Commonly, a low molecular weight biocide is released from a plastic material, in which it is physically entrapped. The 2,4,4′-trichoro-2-hydroxydiphenyl (Triclosan) is the most frequently biocide applied in this strategy [1,2,3]. Further agents used are derivatives of isothiazolone, chlorine releasing *N*-halamines [4], nanosilver [5,6], as well as salts and complexes of metals (typically Cu and Zn) [7,8].

It is also possible to incorporate an antimicrobial moiety to the polymer; these polymers are referred to as intrinsic antimicrobial as the biocidal action results from contact of the polymer and the microorganisms, with no release of the active molecule [9,10]. Typical examples are polymers substituted by quaternary ammonium salts [11,12,13,14,15,16], phosphonium salts [17,18,19,20], and pyridinium groups [21,22,23], respectively.

**Figure 1:**
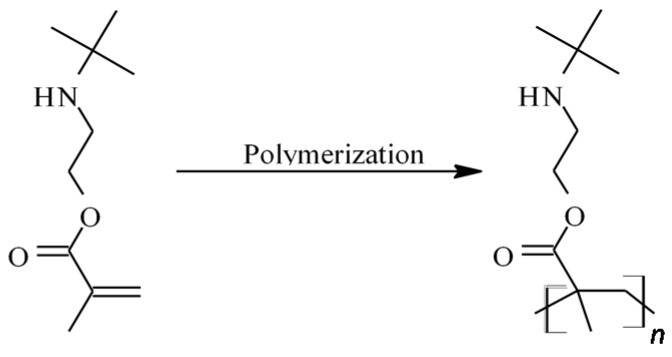
Structure of 2-(*tert*-butylamino) ethyl methacrylate (TBAEMA) and its resulting polymer poly-[2-(*tert*-butylamino) ethyl methacrylate] (poly(TBAEMA)).

Poly[2-(*tert*-butylamino)ethyl methacrylate] (poly(TBAEMA)) is a typical representative of a water-insoluble biocide, its structure is displayed in Figure 1. It was developed in 2001 by Creavis Technologies and Innovation of the Degussa (Marl, Germany). This polymer class was referred as sustainable active microbicidal (SAM-Polymers^®^, Marl, Germany). According to the patent literature, poly(TBAEMA) exhibits inherent biocidal properties and has shown potential application in antifouling paints and coatings [24,25,26,27] or denture base acrylic resins [28,29]. The antimicrobial activity is caused by the pendant bulky secondary amine of the methacrylate backbone, without the need for quaternization, as is the case for other amine-containing polymers [30]. It must be noted that, although poly(TBAEMA) has high antimicrobial activity and low toxicity, the parent monomer shows no antimicrobial effect even at higher concentrations. This finding suggests that the antimicrobial action is closely related to the molecular structure and conformation of the polymer chains [30]. It has been reported that the solubility of poly(TBAEMA) in water (at pH = 7) is lower than 3.0 mg/L. This renders the biocide especially useful for construction materials designed to be in contact with water, since a very low leachability of poly(TBAEMA) from polymer blends and compounds can be expected [31]. A major drawback of poly(TBAEMA) is the low glass transition temperature (*T*_G_) of 40 °C which limits the application as the surface becomes sticky at elevated temperatures. Other weak points are: high water uptake and a tendency for hydrolysis which yields 2-*tert*-butylamino-ethanol. The aim of this study was the development of a new monomer for this class of SAM^®^-polymers, especially raising the *T*_G_ reducing water uptake and avoiding hydrolysis [32].

## 2. Results and Discussion

### 2.1. Synthesis of Aminomtehylstyrenes

**Figure 2:**
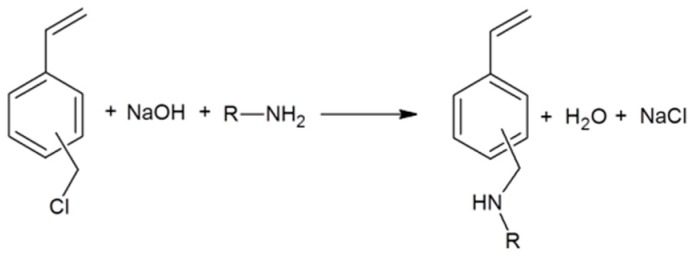
Reaction scheme aminomethylstyrene synthesis.

**Figure 3:**
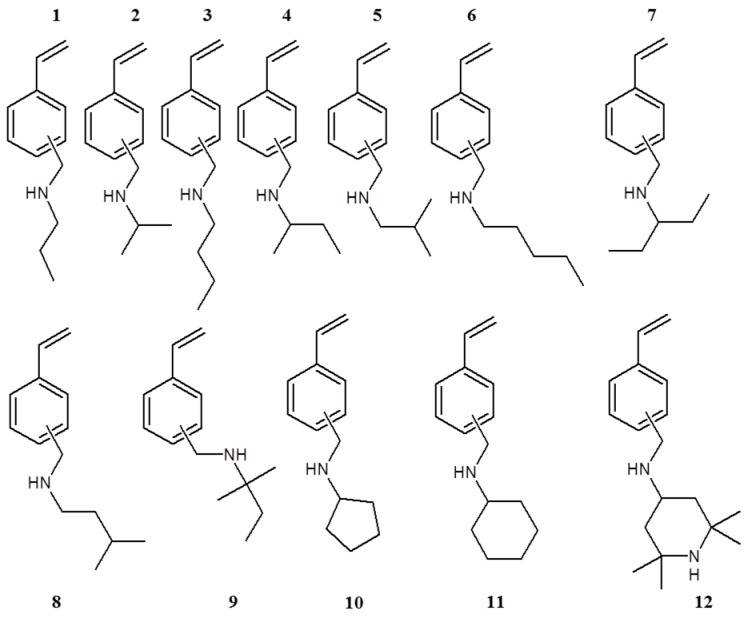
Structures of the alkylaminomethylstyrenes used for screening purpose.

In order to screen a wider variety of different polymers for their glass transition temperatures, a series of aminomethylstyrenes was synthetized using a mixture of meta- and para-Isomers of vinylbenzyl chloride (VBC) and primary amines as starting materials (Figure 2). The reaction proceeds via an S_N_2 mechanism. In order to eliminate salt formation of the secondary amine an excess of sodium hydroxide (NaOH) was used. For a high conversion and short reaction times a twofold excess of amine was applied. The reaction can be carried out under mild conditions due to the high reactivity of the benzyl chloride moiety. Success of the reaction was confirmed by GC-MS and Infrared spectroscopy (IR). The yields were moderate to good (65%–80%), depending on the amine used. The structures of the alkylaminomethylstyrenes are displayed in Figure 3.

The monomers were polymerized as described in Section 3.1. Success of the polymerization was confirmed by IR, where the vinyl bands (e.g., 1630, 988, 903 cm^−1^) vanished after polymerization of the mlkylaminomethylstyrenes. The polymers synthetized from monomers **1**–**6**, **8** and **12** showed a pronounced solubility in water, and were very soft and sticky. In contrast the polymers resulting from the polymerization of the monomers **7** and **9**–**11**, were insoluble in water and isopropyl alcohol (5% *v*/*v*). Additionally, they were more rigid and less sticky than the others. Determination of the glass transition temperature of the polymers formed by polymerization of the monomers **1**–**12** was not possible within the range of 25–160 °C, as no melting peak was observed. Most likely *T*_G_ is below room temperature. The polymers of the monomers **1**–**12** were not suited for technical application because of the softness and stickiness in combination with a *T*_G_ below room temperature and the water solubility of some of them. The poly(*tert*-pentylaminomethylstyrole) (prepared from **9**) had the best characteristics observed in this study so far. Thus, the *tert*-butyl derivative (poly(TBAMS)) may be a candidate that has the desired properties.

### 2.2. Synthesis of N-(1,1-Dimethylethyl)-ethenyl-benzenamine (TBAMS)

**Figure 4:**
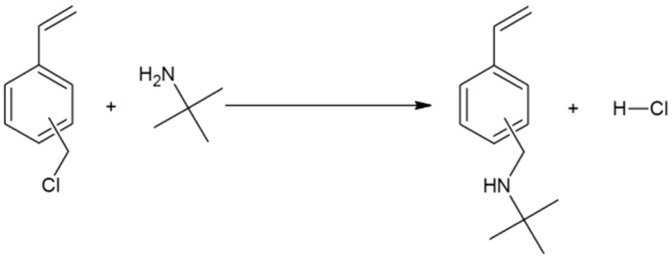
Reaction scheme of 2-(*tert*-butylamino) methylstyrene (TBAMS) synthesis.

**Figure 5:**
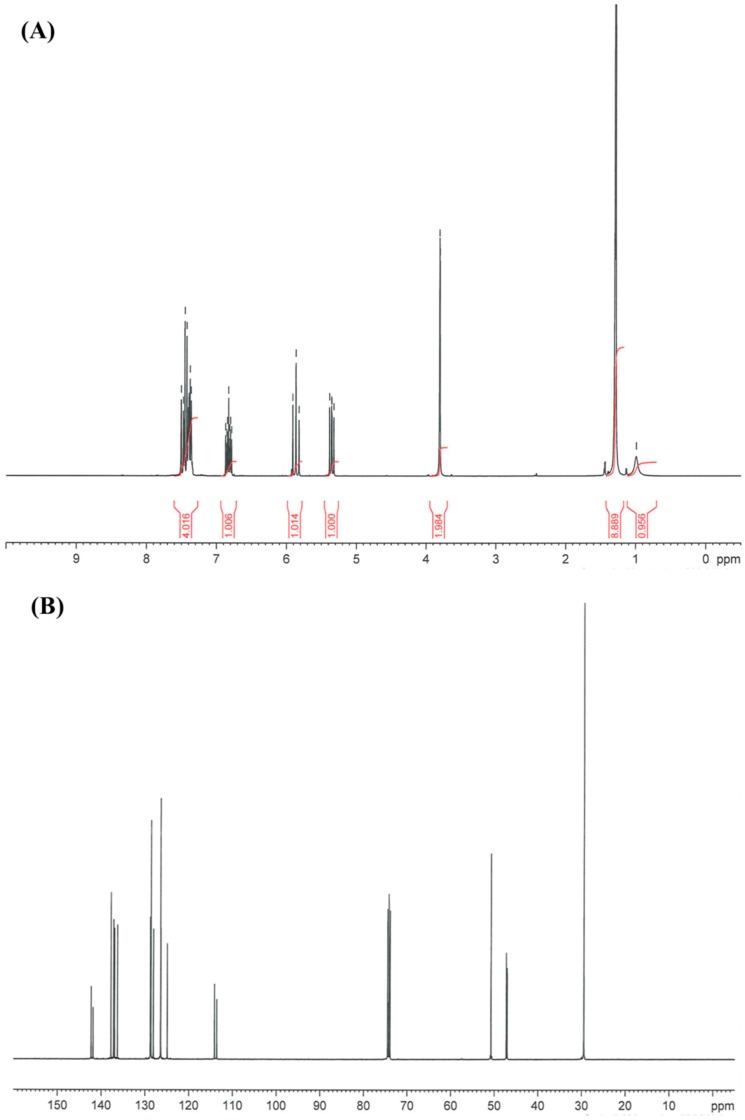
(**A**) ^1^H NMR of TBAMS; (**B**) ^13^C NMR of TBAMS.

The general procedure for the synthesis of the alkylaminomethylstyrenes was optimized for higher yields employing a 3-fold excess of *tert*-butalymine and increasing the reaction temperature to 70 °C (Figure 4). Success of the reaction was confirmed by GC-MS, APCI-MS, IR and NMR. The GC-MS showed two new peaks corresponding to the meta- and para-isomers of TBAMS (57% meta, 43% para), whereas the VBC signals, resulting from the starting material, were very small (3% of the total area in GC-MS). The EI-MS of the product peaks exhibit the molecular ion at 189 *m*/*z*. The signal at 174 *m*/*z* be rationalized a methyl loss, whereas the 117 *m*/*z* can be interpreted as a 4-(ethenyl phenyl)methylium ion that was formed by the loss of the *tert*-butyl amine moiety from the parent ion. The mass peak at 57 *m*/*z* corresponds to the *tert*-butyl group. The infrared spectrum showed at 3306 cm^−1^ a weak band that can be addressed as the *ν*(NH) vibration. In the APCI^+^ mass spectrum the protonated pseudo molecular ion at *m*/*z* 190 [M + H]^+^ was observed. The major fragments are at *m*/*z* 174 represents the (M-CH_4_)^+^, *m*/*z* 132 the (M-*tert*-butyl)^+^ (only observed after collision fragmentation of the isolated *m*/*z* 190) and *m*/*z* 117 for the loss of *tert*-butylamin. The structure was further confirmed by ^1^H and ^13^C NMR (Figure 5).

### 2.3. Synthesis of TBAMS Hydrochloride

For elemental analysis and further characterization of TBAMS the hydrochloride was prepared. The elemental analysis showed a good match of calculated C/N/H-ratios and experimentally determined values. The IR spectrum contains structural features (e.g., the bands at 2742, 2667, 2623 cm^−1^, *etc.*) that can be associated to the ammonium group in the hydrochloride. If the TBAMS hydrochloride is analyzed by GC-MS, the salt decomposes to HCl and TBAMS at the high temperature of the injector. Accordingly TBAMS was observed in the GC-MS chromatogram instead of TBAMS hydrochloride. As mentioned in Section 2.1 traces of VBC were present even in the distilled TBAMS, because the difference of the boiling points of VBC and TBAMS is too small to allow a quantitative separation in a single distillation step. As no residual VBC was present in the GC-MS analysis of TBAMS hydrochloride, the hydrochloride preparation offers a facile way to purify TBAMS.

### 2.4. Preparation of Poly(TBAMS)

**Figure 6:**
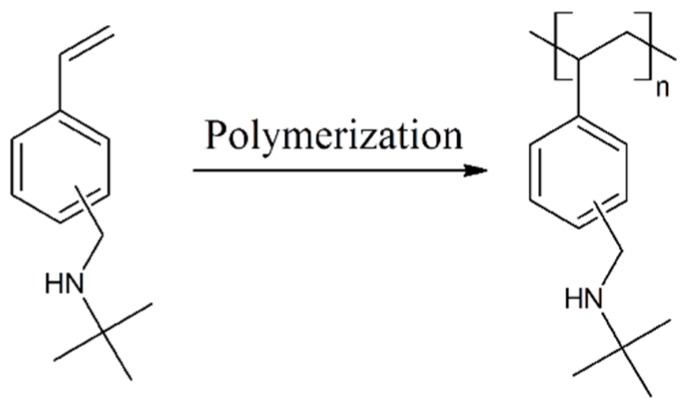
Structure of 2-(*tert*-butylamino) methylstyrene (TBAMS) and its resulting polymer.

**Figure 7:**
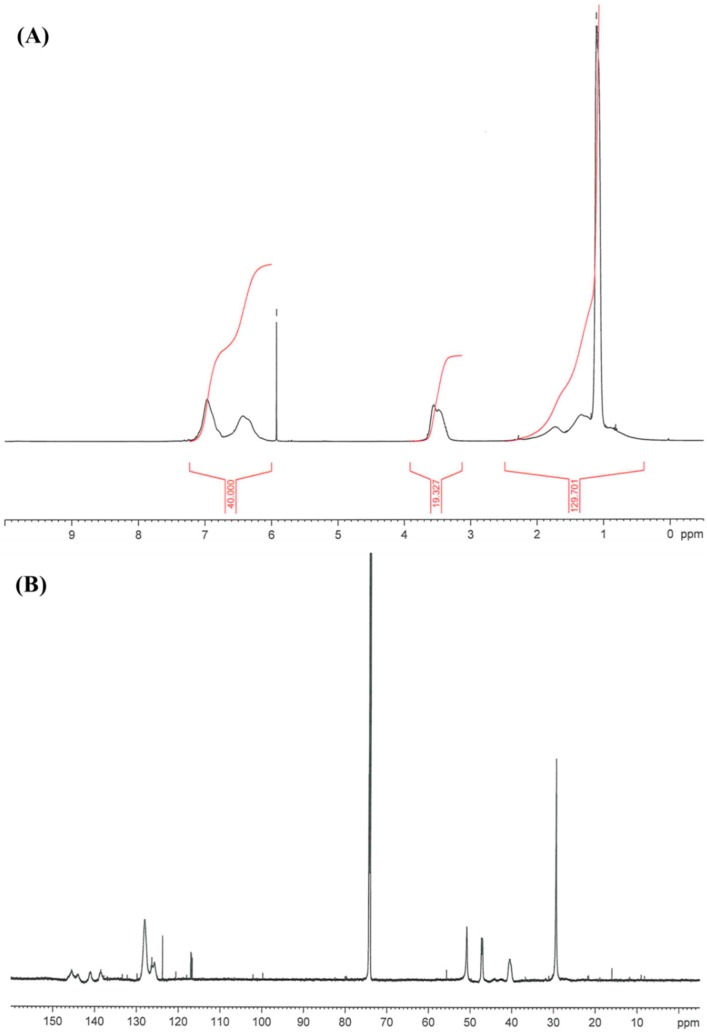
(**A**) ^1^H NMR of poly(TBAMS); (**B**) ^13^C NMR of poly(TBAMS).

Success of the polymerization was confirmed by ^1^H and ^13^C NMR (Figure 7). First experiments to polymerize TBAMS in bulk resulted in small molecular weight polymers. Therefore it was necessary to increase the molecular weight to an acceptable level for technical application. Accordingly polymerization of TBAMS was carried out in ethanol using 2,2′-azobisisobutyronitrile (AIBN) as initiator(Figure 6). The molecular weight (*M*w) was raised to about 185 kDa in typical experiments. The polydispersity (*M*_W_/*M*n) of 5.2 is rather high. A pronounced polydispersity (2.5–3) is typical for AIBN initiated radical reactions. The high polydispersity observed in the poly(TBAMS) polymer can be an indication, that grafting occurs during polymerization. Probably chain transfer occurs at the benzylic position of poly(TBAMS) and TBAMS. Poly(TBAMS) possesses a *T*_G_ of about 68 °C, which is considerably higher than the *T*_G_ of poly(TBAEMA). The poly(TBAMS) was soluble in ethanol and formed uniform, colorless and transparent films upon solvent evaporation. Glass transition temperatures can be further be adjusted by copolymerization to about 160 °C without the loss of the antimicrobial activity. The copolymer was synthetized using 42 mol % TBAMS, 42 mol % 4-vinylpyridine and 16 mol % 4-vinylbenzoic acid. The log reduction of this copolymer was 3.5 for *S. aureus.*

Chitosan is a well-studied intrinsic antimicrobial polymer, which is a possible candidate for technical application. The glass transition temperature varies over a broad range between 61 and 203 °C, depending on molecular weight, degree of deacetylation and residual water content [33,34,35]. A drawback of chitosan is the wet processing of the polymer to form films or blends with other polymers. Depending on the mode of preparation, a high water uptake and swelling in aqueous media is observed. The poly(TBAMS) has a lower water uptake of approximately 3%. Another intrinsic antimicrobial polymer with a *T*_G_ of 218 °C is a poly(styrene-alt-maleic anhydride)-4-aminophenol conjugate [36]. The antimicrobial properties of this polymer regarding the reduction of *E. coli* and *S. aureus* are good but cannot be directly compared to poly(TBAMS) as different methods were used to determine the reduction rates. Methylacrylamide based polymers and copolymers described by Dizman *et al.* also had comparingly high glass transition temperature of 169 °C for the homopolymer [37]. Unfortunately only the quaternized polymers, that were water soluble and had no observable *T*_G_, exhibited good antimicrobial properties.

### 2.5. Laboratory Tests According to JIS Z 2801

The antimicrobial activity of the polymer was analyzed by the test method JIS Z 2801:2000. The test method is based on the comparison of bacterial counts on reference and sample material after inoculation and incubation. The initial bacterial concentration of *S. aureus* determined on references samples without incubation was 1.6 × 10^5^ CFU/mL and for *E. coli* was 4 × 10^5^ CFU/mL.

The arithmetic mean of bacterial counts of *S. aureus* on reference dishes is 5.5 log_10_ CFU/mL and for *E. coli* is 6.5 log_10_ CFU/mL after 2 h incubation time at 35 °C. Bacterial counts of *S. aureus* and *E. coli* on poly(TBAMS) were below the detection limit of 1 log_10_ CFU/mL and show a reduction level of 4.5 log_10_ units for *S. aureus* and 5.5 log_10_ units for *E. coli* respectively. Thus, the antimicrobial activity is proven against *S. aureus* and *E. coli* on poly(TBAMS). Poly(TBAMS) shows excellent antimicrobial properties towards the investigated microorganisms.

### 2.6. Incubation of Poly(TBAMS) in E. coli Culture for Scanning Electron Microscopy (SEM) Imaging

**Figure 8:**
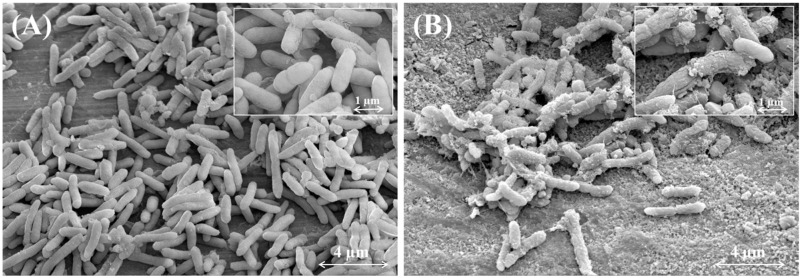
(**A**) *E. coli* after 12 h incubation in blank petri dish; (**B**) *E. coli* after 12 h incubation in petri dish coated with poly(TBAMS).

SEM was applied in order to investigate morphological changes of bacteria in contact with poly(TBAMS). In the case of the gram negative *E. coli* after 12 h of contact pronounced morphological changes were observed, whereas cells in the blank petri dishes remained unchanged. Furthermore the cells show a exudate of fibrous and granular material after incubation with poly(TBAMS). The surface of treated bacteria appears to be wrinkled and rough if compared with untreated cells (Figure 8). Additionally, a lot of debris was observed in the treated *E. coli*. Most likely the debris was formed due the lysis of cells.

The SEM images gave striking evidence that the mode of action of poly(TBAMS) is most likely due to disruption of cell walls. Very similar observations, concerning the morphological changes, have been made in the case of poly(TBAEMA) grafted on polyethylene [38], a poly(*N*,*N*-dimethyl-aminomethylstyrene) bonded to a polystyrene fiber [39] and water soluble β-Chitosan [40].

## 3. Experimental Section

All chemicals were of analytical grade or higher. Vinylbenzyl chloride was purchased from Dow chemicals as a mixture of isomers (57% meta, 43% para).

Melting points were recorded on a SPM30 (Stuart, Staffordshire, UK) using open capillaries. GC-MS were recorded on a QP2010plus (Shimadzu, Duisburg, Germany) using a FS supreme 5 column (30 m, ID 0.25 mm, film 0.25 mm). Detection was performed at 70 eV in EI mode. APCI^+^-MS were recorded on a Bruker esquire 4000 (Bruker Daltonics, Bremen, Germany) instrument. Infrared spectra were recorded on a spectrum one (Perkin Elmer, Waltham, MA, USA) spectrometer using ATR technique. Differential scanning calorimetry analysis was carried out on a DSC821 (Mettler Toledo, Greifensee, Swiss) system. ^1^H NMR and ^13^C NMR spectra were acquired on a Bruker Avance III NMR (Bruker BipSpin, Rheinstetten, Germany) spectrometer with an observing frequency of 400 MHz for ^1^H and 100 MHz for ^13^C, respectively. The samples were measured on the delta (δ) scale and are referenced to Trichloroethane (^1^H δ 5.93 ppm, ^13^C δ 74.2 ppm) that was used as solvent.

### 3.1. Synthesis of Aklylaminomethylstyrenes

In a 1 L round bottom flask equipped with a reflux condenser and dripping funnel there were placed H_2_O (200 mL) and NaOH (42 g, 1.05 mol). After complete dissolution of the NaOH the solution was cooled to 25 °C the amine (1.05 mol) was added. The apparatus was put under inert atmosphere by purging with nitrogen. The heterogeneous mixture was heated to 70 °C using an external oil bath and chloromethylstyrene (53.42 g, 0.35 mol) in THF (150 mL) was added drop wise over the course of 75 min. After the addition was complete the reaction was stirred au 70 °C for additional 24 h. The organic phase was separated, dried over NaSO_4_ and filtered through a sintered glass funnel. Volatiles were removed under reduced pressure (30 mbar, 40 °C). Further workup was performed using fractional distillation.

Propyaminomethylstyrene (**1**): Distillation: 135–140 °C, 20 mbar. Yield: 41.57g (65%). Purity: 96% (GC/MS, total area). Analysis: EI-MS: M^+^ 175 *m*/*z*, 146 *m*/*z*, 117 *m*/*z*, 91 *m*/*z*, 58 *m*/*z*, 39 *m*/*z*. IR: 3309 cm^−1^·w, 3087 cm^−1^·w, 3006 cm^−1^·w, 2953 cm^−1^·m, 2930 cm^−1^·m, 2873 cm^−1^·m, 1629 cm^−1^·m, 1602 cm^−1^·w, 1582 cm^−1^·w, 1510 cm^−1^·m, 1456 cm^−1^·s, 1120 cm^−1^·s, 989 cm^−1^·s, 903 cm^−1^·s, 796 cm^−1^·s, 712 cm^−1^·s.

Isopropylaminomethylstyrene (**2**): Distillation: 85–93 °C, 1 mbar. Yield: 52.82 g (81%). Purity: 95% (GC/MS, total area). Analysis: EI-MS: M^+^ 175 *m*/*z*, 160 *m*/*z*, 117 *m*/*z*, 91 *m*/*z*, 65 *m*/*z*, 30 *m*/*z*.

IR: 3310 cm^−1^·w, 3087 cm^−1^·w, 2963 cm^−1^·s, 2869 cm^−1^·m, 2825 cm^−1^·w, 1630 cm^−1^·m, 1602 cm^−1^·w, 1582 cm^−1^·w, 1510 cm^−1^·m, 1468 cm^−1^·m, 1439 cm^−1^·m, 1405 cm^−1^·m, 1379 cm^−1^·s, 1337 cm^−1^·m, 1171 cm^−1^·s, 1124 cm^−1^·m, 1071 cm^−1^·m, 989 cm^−1^·s, 903 cm^−1^·s, 828 cm^−1^·s, 796 cm^−1^·s, 712 cm^−1^·s.

Butylaminomethylstyrene (**3**): Distillation 150–153 °C, 20 mbar. Yield: 46.06 g (66%). Purity: 96% (GC/MS, total area). Analysis: EI-MS: M^+^ 189 *m*/*z*, 146 *m*/*z*, 117 *m*/*z*, 91 *m*/*z*, 65 *m*/*z*, 29 *m*/*z*.

IR: 3309 cm^−1^·w, 3807cm^−1^·w, 3007 cm^−1^·w, 2955 cm^−1^·m, 2927 cm^−1^·w, 1582 cm^−1^·w, 510 cm^−1^·m, 1456 cm^−1^·s, 1405 cm^−1^·m, 1377 cm^−1^·w, 1120 cm^−1^·s, 988 cm^−1^·s, 903 cm^−1^·s, 825 cm^−1^·s, 796 cm^−1^·s, 712 cm^−1^·s.

Sec-Butylaminomethylstyrole (**4**): Distillation: 140–145 °C, 21 mbar. Yield: 53.21g (76%). Purity: 95% (GC/MS, total area). Analysis: EI-MS: M^+^ 189 *m*/*z*, 174 *m*/*z*, 160 *m*/*z*, 117 *m*/*z*, 91 *m*/*z*, 65 *m*/*z*, 39 *m*/*z*.

IR: 3320 cm^−1^·w, 3087 cm^−1^·w, 2961 cm^−1^·s, 2928 cm^−1^·m, 2875 cm^−1^·m, 1630 cm^−1^·m, 1602 cm^−1^·w,1528 cm^−1^·w, 1510 cm^−1^·m, 1462 cm^−1^·s, 1405 cm^−1^·m, 1373 cm^−1^·m, 1347 cm^−1^·m, 1166 cm^−1^·m, 1079 cm^−1^·m, 988 cm^−1^·s, 903 cm^−1^·s, 828 cm^−1^·s, 796 cm^−1^·s, 712 cm^−1^·s.

Isobutylaminomethylstyrole (**5**): Distillation: 145–14 °C, 24 mbar. Yield: 51.55 g (74%). Purity: 95% (GC/MS, total area). Analysis: EI-MS: M^+^ 189 *m*/*z*, 146 *m*/*z*, 117 *m*/*z*, 91 *m*/*z*, 65 *m*/*z*, 41 *m*/*z*.

IR: 3221 cm^−1^·w, 3087 cm^−1^·w, 3007 cm^−1^·w, 2953 cm^−1^·s, 2870 cm^−1^·m, 2809 cm^−1^·m, 1630 cm^−1^·m, 1602 cm^−1^·w, 1582 cm^−1^·w, 1510 cm^−1^·m, 1462 cm^−1^·s, 1444 cm^−1^·s, 1405 cm^−1^·m, 1385 cm^−1^·m, 1365 cm^−1^·m, 1109 cm^−1^·s, 988 cm^−1^·s, 903 cm^−1^·s, 796 cm^−1^·s, 722 cm^−1^·s.

Pentylaminomethylstyrole (**6**): Distillation: 117–123 °C, 1 mbar. Yield: 56.42 g (76%). Purity: 96% (GC/MS, total area). Analysis: EI-MS: M^+^ 203 *m*/*z*, 146 *m*/*z*, 117 *m*/*z*, 43 *m*/*z*, 29 *m*/*z*.

IR: 3309 cm^−1^·w, 3087 cm^−1^·w, 3007 cm^−1^·w, 2954 cm^−1^·s, 2915 cm^−1^·s, 2868 cm^−1^·m, 2816 cm^−1^·m, 1630 cm^−1^·m, 1602 cm^−1^·w, 1582 cm^−1^·w, 1510 cm^−1^·m, 1463 cm^−1^·s, 1405 cm^−1^·m, 1383 cm^−1^·m, 1366 cm^−1^·m, 1110 cm^−1^·s, 988 cm^−1^·s, 903 cm^−1^·s, 795 cm^−1^·s, 712 cm^−1^·s.

3-Pentylaminomethylstyrole (**7**): Distillation: 120–123 °C, 1 mbar. Yield: 59.65 g (76%). Purity: 95% (GC/MS, total area). Analysis: EI-MS: M^+^ 203 *m*/*z*, 146 *m*/*z*, 117 *m*/*z*, 91 *m*/*z*, 41 *m*/*z*.

IR: 3336 cm^−1^·w, 3088 cm^−1^·w, 2960 cm^−1^·m, 2929 cm^−1^·m, 2873 cm^−1^·m, 1630 cm^−1^·m, 1602 cm^−1^·w, 1582 cm^−1^·w, 1510 cm^−1^·m, 1549 cm^−1^·s, 1405 cm^−1^·m, 1379 cm^−1^·m, 1159 cm^−1^·m, 1085 cm^−1^·m, 989 cm^−1^·s, 903 cm^−1^·s, 796 cm^−1^·s, 713 cm^−1^·s.

Isopentylaminomethylstyrole (**8**): Distillation: 140–145 °C, 9 mbar. Yield: 50.93 g (76%). Purity: 96% (GC/MS, total area). Analysis: EI-MS: M^+^ 203 *m*/*z*, 146 *m*/*z*, 117 *m*/*z*, 41 *m*/*z*.

IR: 3309 cm^−1^·w, 3087 cm^−1^·w, 3007 cm^−1^·w, 2954 cm^−1^·s, 2915 cm^−1^·s, 2868 cm^−1^·m, 2816 cm^−1^·m, 1630 cm^−1^·m, 1602 cm^−1^·w, 1582 cm^−1^·w, 1510 cm^−1^·m, 1463 cm^−1^·s, 1405 cm^−1^·m, 1387 cm^−1^·m, 1366 cm^−1^·m, 1110 cm^−1^·s, 988 cm^−1^·s, 903 cm^−1^·s, 796 cm^−1^·s, 712 cm^−1^·s.

*Tert*-pentylaminomethylstyrole (**9**): Distillation: 105–110 °C, 1 mbar. Yield: 55.12 g (74%). Purity: 96% (GC/MS, total area). Analysis: EI-MS: M^+^ 203 *m*/*z*, 188*m*/*z*, 174 *m*/*z*, 117 *m*/*z*, 91 *m*/*z*, 42 *m*/*z*.

IR: 3320 cm^−1^·w, 3087 cm^−1^·w, 2963 cm^−1^·s, 2887 cm^−1^·s, 1630 cm^−1^·m, 1602 cm^−1^·w, 1582 cm^−1^·w, 1510 cm^−1^·m, 1460 cm^−1^·s, 1405 cm^−1^·m, 1380 cm^−1^·m, 1363 cm^−1^·m, 1289 cm^−1^·s, 1201 cm^−1^·s, 1093 cm^−1^·s, 988 cm^−1^·s, 903 cm^−1^·s, 827 cm^−1^·s, 796 cm^−1^·s, 712 cm^−1^·s.

Cyclopentylaminomethylstyrole (**10**): Distillation: 132–140 °C, 1 mbar. Yield: 51.2 g (70%). Purity: 96% (GC/MS, total area). Analysis: EI-MS: M^+^ 201 *m*/*z*, 172*m*/*z*, 158 *m*/*z*, 117 *m*/*z*, 91 *m*/*z*, 84 *m*/*z*, 65 *m*/*z*, 41 *m*/*z*.

IR: 3315 cm^−1^·w, 3086 cm^−1^·w, 3006 cm^−1^·w, 2924 cm^−1^·s, 2851 cm^−1^·s, 1630 cm^−1^·m, 1602 cm^−1^·w, 1582 cm^−1^·w, 1510 cm^−1^·m, 1448 cm^−1^·s, 1405 cm^−1^·m, 1360 cm^−1^·m, 1346 cm^−1^·m, 1121 cm^−1^·s, 988 cm^−1^·s, 903 cm^−1^·s, 826 cm^−1^·s, 796 cm^−1^·s, 712 cm^−1^·s.

Cyclohexylaminomethylstyrole (**11**): Distillation: 135–137 °C, 1 mbar. Yield: 62.75 g (80%). Purity: 95% (GC/MS, total area). Analysis: EI-MS: M^+^ 215 *m*/*z*, 172*m*/*z*, 158 *m*/*z*, 117 *m*/*z*, 98 *m*/*z*, 91 *m*/*z*, 55 *m*/*z*, 41 *m*/*z*.

IR: 3310 cm^−1^·w, 3086 cm^−1^·w, 3006 cm^−1^·w, 2924 cm^−1^·s, 2851 cm^−1^·s, 1630 cm^−1^·m, 1602 cm^−1^·w, 1581 cm^−1^·w, 1510 cm^−1^·m, 1448 cm^−1^·s, 1405 cm^−1^·s, 1360 cm^−1^·m, 1346 cm^−1^·m, 1124 cm^−1^·s, 988 cm^−1^·s, 903 cm^−1^·s, 826 cm^−1^·s, 795 cm^−1^·s, 712 cm^−1^·s.

Tetramethylpiperidinaminomethylstyrole (**12**): Distillation: 165–170 °C, 2 mbar. Yield: 60.60 g (60%). Purity: 95% (GC/MS, total area). Analysis: EI-MS: M^+^ 272 *m*/*z*, 257 *m*/*z*, 215 *m*/*z*, 173 *m*/*z*, 155 *m*/*z*, 117 *m*/*z*, 98 *m*/*z*, 58 *m*/*z*, 41 *m*/*z*.

IR: 3305 cm^−1^·w, 3085 cm^−1^·w, 2954 cm^−1^·s, 2917 cm^−1^·s, 1630 cm^−1^·m, 1601 cm^−1^·w, 1582 cm^−1^·w, 1510 cm^−1^·m, 1454 cm^−1^·m, 1374 cm^−1^·s, 1363 cm^−1^·s, 1237 cm^−1^·s, 1197 cm^−1^·s, 1107 cm^−1^·s, 989 cm^−1^·s, 904 cm^−1^·s, 826 cm^−1^·m, 795 cm^−1^·s, 712 cm^−1^·s.

### 3.2. Polymerization of the Aklylaminomethylstyrenes

In a 250 mL round bottom flask the corresponding monomer (145.8 mmol) were dissolved in ethanol (60 mL), a solution of AIBN (0.37 g) in 2-butanone (10 mL) was added. The apparatus was put under inert atmosphere by purging with nitrogen. The contents of the flask were heated under constant stirring (300 rpm) to 60 °C with internal temperature control in a silicon oil bath for 48 h. The polymer was precipitated by slowly dripping the contents of the flask in to a stirred bath of 5 L H_2_O. The solids were removed by filtration. Removal of residual monomers and oligomers was accomplished by boiling the polymer (20 g) in aqueous isoporopyl alcohol (100 mL, 5% (*v*/*v*)). Finally, the polymer was dried in a vacuum drying cabinet (Binder VD23, Tuttlingen, Germany) at 140 °C, 1 mbar until no more change in polymer mass was observed. Water soluble polymers were directly dried *in vacuo* after removal of volatiles (40 °C, 1 mbar) without prior precipitation in water.

### 3.3. Synthesis of TBAMS

In a 1 L three neck round bottom flask, equipped with reflux condenser, dripping funnel and magnetic stir bar H_2_O (300 mL) and NaOH (36 g, 0.9 mol) were placed. The solution was cooled down to room temperature and *tert*-butyl amine (65.82 g, 0.9 mol) was added. The apparatus was put under inert atmosphere by purging with nitrogen. The contents of the flask were heated to 60 °C with internal temperature control in a silicon oil bath. Vinylbenzyl chloride (45.78 g, 0.3 mol) in THF (200 mL) was added in a drop wise manner over the course of 4 h. Stirring and heating was continued over 20 h. After this the organic phase was separated using an extraction funnel. It was washed with H_2_O (2 × 50 mL) and dried over MgSO_4_. TBAMS was isolated by fractional distillation (105–114 °C, 2 mbar) as colorless oil with a high refractive index. The yield was 55.65 g (96%). Purity was 97% (GC-MS, total area).

Analysis: EI-MS: M^+^ 189 *m*/*z*, 1.08% ;174 *m*/*z*, 26.23%; 117 *m*/*z* 100.00%; 91 *m*/*z*, 9.06%; 57 *m*/*z*, 3.76%; 41 *m*/*z*, 6.92%. IR: 3306 cm^−1^·w, 3088 cm^−1^·w, 2963 cm^−1^·s, 1630 cm^−1^·m, 1510 cm^−1^·m, 1479 cm^−1^·s, 1307 cm^−1^·m, 1228 cm^−1^·s; 1211 cm^−1^·s, 988 cm^−1^·s, 903 cm^−1^·s, 827 cm^−1^·s, 797 cm^−1^·s, 712 cm^−1^. ^1^H NMR: δ 0.99 ppm (s, 1H, NH), δ 1.30 ppm (s, 9H, –(CH_3_)_3_), δ 3.80 ppm (s, 2H, –CH_2_–NH), δ 5.35 ppm and δ 5.87 ppm (t, 2H, –CH=CH_2_), δ 6.85 ppm (m, 1H, –CH=CH_2_ ), δ 7,42 ppm (m, 4H, aromatic); ^13^C NMR: δ 29.5 ppm (C–(CH_3_)), δ 47, 47.2 ppm (–CH_2_–NH), δ 50.7 ppm (NH–C(CH_3_)_3_), δ 114.1.7, 113 ppm (–CH=CH_2_), δ 124.9, 126.4 128.1, 128.8 ppm (aromatic CH), δ 126.4 128.6 ppm (aromatic CH of para isomer), δ 136.3, 136.9 137.2, 137.8 ppm (Quart-aromatic C), δ 141.8 142.3 ppm (–CH=CH_2_).

### 3.4. Preparation of TBAMS Hydrochloride

In a 500 mL round bottom flask TBAMS (70 g, 0.369 mol) in Acetone (150 mL) was placed. The flask was nestled in an ice bath and stirred magnetically. A slow stream of dry HCl (from NaCl and conc. H_2_SO_4_) was introduced via a glass capillary in to the solution until the solution was slightly acidic. Stirring was continued for 2 h. After precipitation of the hydrochloride at −20 °C, the crystals were removed by filtration, washed with ice cold acetone (2 × 10 mL) and dried in vacuum. The crystals were recrystallized from isopropyl alcohol/toluene (10 mL/150 mL). After cooling to −20 °C and drying in vacuum TBAMS℘HCl (66.7 g, 80% yield) was obtained.

Analysis: melting point: 172–174 °C decomp.; IR: 2947 cm^−1^·s; 2745 cm^−1^·s, 2430 cm^−1^·m; 1629 cm^−1^·m; 1589 cm^−1^·s; 1479 cm^−1^·m; 1447 cm^−1^·s; 1402 cm^−1^·s; 1367 cm^−1^·s; 1241 cm^−1^·m; 1198 cm^−1^·s; 985 cm^−1^·s; 924 cm^−1^·s; 808 cm^−1^·s; 711 cm^−1^·s; 570 cm^−1^·s. Elemental analysis: found: C 69.1%; H 8.99%, N 6.2%, calculated for C_13_H_20_ClN: C 69.1%, H 8.9%, N 6.2%.

### 3.5. Preparation of Poly(TBAMS)

TBAMS (27 g, 0.14 mol) was placed in a 500 mL two-neck round-bottom flask equipped with a reflux condenser (Duran glass, Germany). In a 30 mL glass crimp-top vial 2,2′-Azobisisobutyronitrile (AIBN, 0.37 g) was dissolved in 2-butanone (10 mL). This initiator solution was added to the monomer, followed by ethanol (150 mL). The flask was transferred into an oil bath at room temperature (HBR 4 digital, IKA, Staufen, Germany) which was also used to stir the mixture via a magnetic stirrer. The apparatus was flushed with nitrogen (purity 99.99999%) for at least 30 min to ensure an oxygen free atmosphere. The reaction solution was continuously stirred (approx. 300 rpm) during this time. Polymerization was carried out for 5 h at 55 °C and additionally for 22.5 h at 65 °C. After the solution was cooled to room temperature, H_2_O (100 mL) was slowly added via a 250 mL dropping funnel under stirring. After all water was added the stirrer was turned off and the solution stored for 12 h to separate the polymer from the upper ethanol phase which was removed by decantation. The precipitated polymer was dissolved in ethanol (135 mL) and the aforementioned precipitation process was repeated. The liquid phase is then analyzed via TLC (Silicagel 60, MeOH, UV detection). The polymer remained at the start, whereas the Monomer had a retention factor value of approximately 0.5. The purification process was repeated until no more TBAMS can be observed via TLC. In a typical experiment, eight purification steps were necessary. The purified poly(TBAMS) solution was dried in a vacuum drying cabinet (Binder VD23, Tuttlingen, Germany) at 70 °C, 1 mbar for 16 h and milled down using an ultra-centrifugal mill (ZM 200, Retsch, Haan, Germany). A 200 μm sieve was applied, the mill is continuously cooled using liquid nitrogen. Yield: 20 g, 74%.

GPC measurements were performed on a Viscotec GPC max VE2001 (Malvern Instruments, Malvern, United Kingdom) machine equipped with two PSS SD LIN M 5 μm 8 × 300 mm columns (Polymer Standards Services, Mainz, Germany) coupled in series using THF with 0.1% triethylamine as eluent and RI detection. Calibration was done with a Polystyrene standard (474-2520000 Da) (Polymer Standards Services, Mainz, Germany).

Analysis: *T*_G_: 68° C ;GPC: Mw: 185588 Da, Mn: 3562 Da, *M*_W_/*M*n: 5.2; IR: 2960 cm^−1^·s, 2921 cm^−1^·s, 1605 cm^−1^·m, 1443 cm^−1^·s, 1360 cm^−1^·s, 1228 cm^−1^·s, 1088 cm^−1^·m, 1019 cm^−1^·m, 810 cm^−1^·m, 792 cm^−1^ cm, 704 cm^−1^; ^1^HNMR: δ 0.4–2.5 ppm (–(CH_3_)_3_, NH, CH and CH_2_ from polymer backbone), δ 3.10–3.90 ppm (–CH_2_–NH), δ 6.00–7.2 ppm (aromatic H); ^13^C NMR: δ 29.4 ppm (–(CH_3_)_3_), δ 40.4 ppm (CH_2_ from polymer backbone), δ 41.5–45.5 ppm (CH from polymer backbone), δ 47.0–47.3 ppm (–CH_2_–NH), δ 50.8 ppm (–(CH_3_)_3_), δ 124.0–129.0 ppm (aromatic CH) δ138.8, 141.0, 143.9, 145.5 ppm (Quart-aromatic C).

### 3.6. Preparation of Samples for Antimicrobial Assay

For each sample 125 mg of purified polymer was dissolved in 3 mL ethanol under stirring. The solution was then cast in a petri dish without vents (polystyrene, *d* = 90 mm, *h* = 14.2 mm, VWR, Langenfeld, Germany) and dried in a vacuum drying cabinet (Heraeus Vacutherm VT 6025, Thermo Scientific, Waltham, MA, USA) at 70 °C and 2 mbar for 1 h.

### 3.7. Antimicrobial Assessment

The antimicrobial activity of purified poly(TBAMS) was tested according to the Antimicrobial products-Test for antimicrobial activity and efficacy (JIS Z 2801:2000). In each test three poly(TBAMS) samples and three blank petri dishes of the same size were used as reference. *Staphylococcus aureus* ssp. *aureus* (DSM No. 799) and *Escherichia coli* (DSM No. 1576) were applied as test organisms. The inoculum was prepared by transferring a frozen culture to 10 mL nutrient broth (Roth, Karlsruhe, Germany), afterwards the broth was incubated at 37 °C for 24 h. In the beginning of each trial the inoculum was diluted in physiological saline solution with tryptone (Oxoid, Hampshire, UK) to a final concentration of 10^5^ cfu/mL. Samples and references were inoculated with 0.4 mL of this solution. To prevent evaporation and to standardize the contact area, the inoculums were covered loosely by sterile PE films (40 × 40 mm).

Three references were directly washed out with 10 mL soybean casein digest broth with lecithin polysorbat (Roth, Karlsruhe, Germany) to determine the starting concentration. The poly(TBAMS) samples and the remaining references were incubated at 35 °C and 90% humidity for 2 h. Afterwards all samples were washed in a similar manner. Viable counts were determined by counting the colonies on plate count agar (Roth, Karlsruhe, Germany) which were incubated for 48 h at 37 °C.

The value of antimicrobial activity were calculated by subtracting the logarithmic value of viable counts on poly(TBAMS) from the logarithmic value of reference material after inoculation and incubation:

(1) $$log_{10}-Reduction=log_{10}\left(\frac{Ref_{2h}}{poly{\left(TBAMS\right)}_{2h}}\right)$$

whereas cfu_(*T*x, reference)_ = arithmetic mean of bacterial counts on reference material 2 h after inoculation, and cfu_(*T*x, sample)_ = arithmetic mean of bacterial counts on poly(TBAMS) material 2 h after inoculation.

According to the JIS Z 2801:2000, a material can be characterized as antimicrobial, if the calculated log_10_-reduction is ≥2.0 after 24 h at 35 °C.

### 3.8. Incubation of Poly(TBAMS) in Escherichia coli (DSM 1576) Culture for SEM Imaging

In a petri dish containing the poly(TBAMS) film there was added 4.5 mL of an overnight culture of *Escherichia coli* in a complex medium (0.8 g microbiology broth (order No. 1.05443.0500, Merck), 0.84 g NaCl, and 0.1 g Trypton (Applichem, Darmstadt, Germany) and 100 mL using H_2_O, pH 7.4). Blank petri dishes were used as reference. The petri dishes were incubated at room temperature for 12 h. The bacteria suspension was transferred into a 15 mL centrifuge tube, the bacteria were fixed chemically using 2.5 mL of a aqueous 2% glutaraldehyde solution at 4 °C for 3 h. After this the suspension was centrifuged for 10 min at 400 rpm to harvest the bacteria. The supernatant was discarded and the bacteria were dehydrated for 15 min in a series of ethanol/water solutions of increasing ethanol content (30%, 50%, 70% and 90%). The last drying step was repeated two times using absolute ethanol. Ethanol was removed in a vacuum exsiccator at 500 mbar. The pellets were transferred to a SEM sample holder. Gold was deposited on the specimens with an ion spatter. For scanning electron microscopy a Leo 982 “Gemini” (Zeiss, Jena, Germany) microscope was used.

## 4. Conclusions

The preparation and characterization of TBAMS and poly(TBAMS) was described in this study. The synthesis of TBAMS from commercially available starting materials was straightforward and high yielding. Polymerization to poly(TBAMS) generated a new intrinsic antimicrobial polymer with an acceptable glass transition temperature and good film building properties. Excellent antimicrobial action against *E. coli* and *S. aureus* was observed. First insights from the SEM experiments showed that the antimicrobial activity correlates with the disruption of the cell membranes. Further experiments elucidating the applicability of poly(TBAMS) as a antimicrobial additive in compounds are currently underway.
